# Untailored vs. Gender- and Body-Mass-Index-Tailored Skeletal Muscle Mass Index (SMI) to Assess Sarcopenia in Advanced Head and Neck Squamous Cell Carcinoma (HNSCC)

**DOI:** 10.3390/cancers15194716

**Published:** 2023-09-25

**Authors:** Cristina Morelli, Vincenzo Formica, Paolo Bossi, Michela Rofei, Simona Guerriero, Silvia Riondino, Renato Argirò, Noemi Pucci, Tonia Cenci, Luca Savino, Carla G. Rinaldi, Francesco Garaci, Augusto Orlandi, Rolando M. D’Angelillo, Hendrik-Tobias Arkenau, Mario Roselli

**Affiliations:** 1 Medical Oncology Unit, Department of Systems Medicine, Tor Vergata University Hospital, 00133 Rome, Italy; cristina.morelli89@gmail.com (C.M.); rofei.michela@gmail.com (M.R.); simonaguerriero46@gmail.com (S.G.); silvia.riondino@uniroma2.it (S.R.); mario.roselli@uniroma2.it (M.R.); 2 Medical Oncology, Department of Medical and Surgical Specialties, Radiological Sciences and Public Health, “ASST Spedali Civili di Brescia”, University of Brescia, 25123 Brescia, Italy; paolo.bossi@unibs.it; 3 Interventional Radiology Unit, Department of Biomedicine and Prevention, University of Rome “Tor Vergata”, 00133 Rome, Italy; renato.argiro@gmail.com; 4 Neuroradiology Unit, Department of Biomedicine and Prevention, University of Rome “Tor Vergata”, 00133 Rome, Italy; puccinoemi@gmail.com (N.P.); francesco.garaci@uniroma2.it (F.G.); 5 Anatomic Pathology, Department of Biomedicine and Prevention, University of Rome “Tor Vergata”, 00133 Rome, Italy; tonia.cenci@ptvonline.it (T.C.); luca.savino@ptvonline.it (L.S.); orlandi@uniroma2.it (A.O.); 6 Radiation Oncology, Department of Biomedicine and Prevention, University of Rome “Tor Vergata”, 00152 Rome, Italy; carlagermana.rinaldi@ptvonlime.it (C.G.R.); d.angelillo@med.uniroma2.it (R.M.D.); 7 Ellipses Pharma, London W1J 8LG, UK; tobi@ellipses.life

**Keywords:** head and neck cancer, sarcopenia, skeletal muscle mass index

## Abstract

**Simple Summary:**

In this study, we investigated the optimal SMI cutoff to discriminate overall survival (OS) in a cohort of consecutive advanced HNSCC patients. Untailored, BMI-tailored and gender-tailored cutoffs were analyzed, and gender-tailored SMI categorization had the highest prognostic value, which was retained in a multivariate analysis including several HNSCC-specific prognostic factors.

**Abstract:**

(1) Background: Sarcopenia lasting >1 year might be considered a chronic condition in many HNSCC patients. CT-scan-derived Skeletal Muscle Mass Index (SMI) is an established surrogate of sarcopenia; yet, the cut-off reported in the literature (literature-based, lb-SMI < 43.2) is mainly based on the risk of chemoradiotherapy-induced toxicity, and the optimal value to discriminate OS is under-investigated. (2) Methods: The effect on OS of the lb-SMI cutoff was compared with an untailored OS-oriented SMI cutoff obtained in a cohort of consecutive advanced HNSCC patients treated with primary chemoradiotherapy, bio-chemotherapy or chemo-immunotherapy (cohort-specific, cs-SMI cutoff). Gender- and BMI-tailored (gt-SMI and bt-SMI) cut-offs were also evaluated. Cutoff values were identified by using the maximally selected rank statistics for OS. (3) Results: In 115 HNSCC patients, the cs-SMI cutoff was 31.50, which was lower compared to the lb-SMI reported cut-off. The optimal cut-off separately determined in females, males, overweight and non-overweight patients were 46.02, 34.37, 27.32 and 34.73, respectively. gt-SMI categorization had the highest effect on survival (*p* < 0.0001); its prognostic value was independent of the treatment setting or the primary location and was retained in a multivariate cox-regression analysis for OS including other HNSCC-specific prognostic factors (*p* = 0.0004). (4) Conclusions: A tailored SMI assessment would improve clinical management of sarcopenia in chemoradiotherapy-, bio-chemotherapy- or chemo-immunotherapy-treated HNSCC patients. Gender-based SMI could be used for prognostication in HNSCC patients.

## 1. Introduction

Nearly 900,000 new diagnoses of and 500,000 deaths from head and neck squamous cell carcinoma (HNSCC) were recorded in 2020 worldwide, accounting for 9% of all cancer diagnoses and 5% of all cancer deaths [1]. More than 60% of HNSCC are locoregionally advanced or with distant metastases at diagnosis (stage III and IV, respectively) [2]. Moreover, in 15 to 40% of patients, disease recurrence occurs after definitive local treatment [2].

The current standard of care for locally advanced nonsurgical HNSCC patients is radiotherapy plus concomitant cisplatin, while in the case of recurrent disease not amenable to re-irradiation or in case of metastases, first-line systemic therapy with either chemotherapy plus anti-EGFR or anti-Programmed death protein 1 (PD-1) with or without concomitant chemotherapy is administered [3].

HNSCC patients are frequently frail and sarcopenic and require multidisciplinary care that should take into consideration multiple management aspects, such as pain, swallowing and speech dysfunction, nutritional support and psychological distress [3].

Sarcopenia is a syndrome characterized by loss of muscle mass and function that can be physiological and secondary to aging or associated with various chronic diseases such as cancer [4]. It is strongly related to impaired quality of life and depression symptoms [5]. Sarcopenia, found in approximately 20–30% of cancer patients at diagnosis, can emerge as a chronic illness [6,7], i.e., lasting >1 year (https://www.cdc.gov/chronicdisease/about/index.htm, accessed on 15 July 2023), and can be associated with worse surgical outcome and survival, as well as higher treatment-related toxicity [8]. More recently, its prognostic role has been confirmed also in patients treated with immune check-point inhibitors across multiple tumor types [9,10,11]. Computed tomography (CT)-derived skeletal muscle mass index (SMI) was proposed to quantify the total amount of skeletal muscle mass and assess sarcopenia [12]. Information for SMI can be obtained from a single lumbar or cervical CT-slice and this guarantees accessibility and good quality data for possibly all HNSCC patients [13,14]. Wong et al., in a recent meta-analysis including nine retrospective and one prospective studies [15], confirmed that radiologically defined sarcopenia is a negative predictor of OS in HNSCC, independently of the selected CT-slice. Although easily available, specific threshold values of SMI for HNSCC patients are under-investigated [15,16] and the comparability between the analysis at the cervical and lumbar level has been questioned [15,17].

Prado et al. highlighted the importance of body mass index (BMI) and gender in the assessment of sarcopenia [17,18] and recommended that sex-specific cut-offs of SMI should be considered [17]. Importantly, gender differences in relation to sarcopenia have been reported with a higher prevalence of sarcopenic obesity found in men (defined as contemporary presence of overweight and depleted muscle mass) [19].

In the literature, SMI threshold values have been widely applied regardless of cancer type, treatment setting and patient characteristics, without appreciating potential differences in SMI cut-offs, particularly between overweight and non-overweight as well as male vs. female patients with HNSCC.

The aim of the present study was to define the optimal survival-related SMI cut-off in advanced (locoregionally and metastatic) HNSCC patients treated with primary chemoradiotherapy, bio-chemotherapy or chemoimmunotherapy and to evaluate different threshold values based on BMI and gender subgroups.

## 2. Materials and Methods

Patients with histologically confirmed advanced HNSCC and not candidates for surgery were treated between December 2014 and October 2022 at the ‘Tor Vergata’ University Hospital of Rome with definitive cisplatin-based chemoradiotherapy for locally advanced disease, and standard first-line bio-chemotherapy or immuno-chemotherapy for recurrent/metastatic disease was retrospectively reviewed.

Routine demographic and clinical characteristics were collected at baseline: age at diagnosis, gender, alcohol consumption, height (m), weight (kg), BMI (weight to square of height, kg/m^2^), primary tumor site, stage, Charlson Comorbidity Score (CCS) [20], smoking habit, Human Papilloma Virus (HPV) status, Karnofsky Performance Status (KPS) and modified Glasgow Prognostic Score (mGPS) (a score that ranges from 0 to 2 based on the presence/absence of the following adverse factors: albuminemia < 35 g/L and C-reactive protein > 10 mg/L) [21]. Patients were classified as overweight (BMI > 25) or non-overweight (BMI < 25), according to World Health Organization (WHO) classification [22].

**Figure 1:**
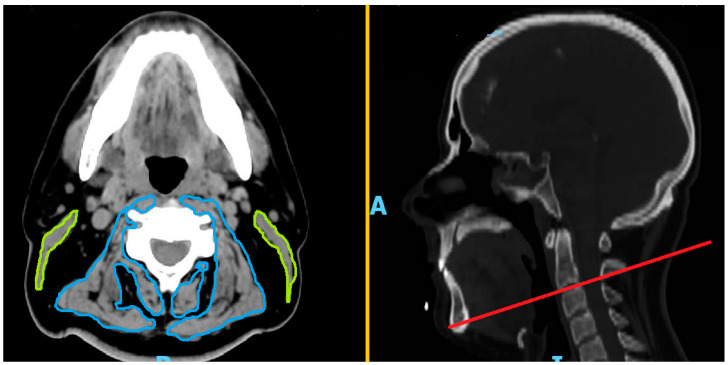
Representative model of C3 CT-scan slice measurement of paravertebral and sternocleidomastoid muscles.

In order to allow SMI measurement, only patients with a pretreatment computed tomography (CT) scan with contrast reconstructed with a 1 mm slice thickness were included in the analysis. SMI was obtained according to standard procedure as follows: using C3 sagittal slice as the landmark and setting Hounsfield units (HU) within −29 HU to +150, corresponding to skeletal muscle density, right and left sternocleidomastoid and paravertebral muscle areas were manually selected and measured by two distinct operators (M.R. and S.G.) with discrepancies resolved by a third operator (R.A.). In Figure 1 a showing the representative SMI calculation, the sum of the selected areas corresponded to the total cross-sectional area (CSA, cm^2^) of the skeletal muscle at C3, according to the method previously published by Swartz et al. [23]. In case of unilateral tumor infiltration of the muscles or surgical removal, the target area was calculated by doubling the area of the contralateral muscles [23]. CSA at L3 was finally derived by CSA at C3 using the equation described by Swartz et al. [23]. The final skeletal muscle index (SMI, cm^2^/m^2^) resulted from the CSA at L3 divided by the square of the height (m^2^).

SMI-based patient categorization in sarcopenic vs. non-sarcopenic (i.e., SMI low vs. high) was performed according to four different methods and the prognostic value of these categorization methods was assessed and compared, with the primary outcome measure being overall survival (OS). OS was defined as the time from the start of the main treatment (chemoradiotherapy or first-line chemotherapy) to the day of death due to any cause or last follow-up visit.

First, sarcopenia was defined a priori as an SMI of less than 43.2, based on the report by Wendrich et al. [16] that was focused on the prediction of chemotherapy dose-limiting toxicity in HNSCC (literature-based SMI, lb-SMI cutoff).

The second method was based on the identification of a prognostic ‘cohort-specific’ SMI (cs-SMI) cutoff. Taking SMI as a continuous variable, maximally selected rank statistics for overall survival (OS) [24] were applied to find the most significant cut-off associated with survival in our specific population.

Patients were then stratified by BMI (<25 vs. >25 Kg/m^2^) and gender (females vs. males), and for each subgroup, a specific SMI cut-off was obtained by using the maximally selected rank statistics for OS. The third and fourth methods were therefore the definition of sarcopenia according to a BMI-tailored (bt) and a gender-tailored (gt) cutoff (i.e., two distinct SMI cutoffs based on BMI, one for overweight and one for non-overweight patients; and two distinct cutoffs based on gender, one for males and one for females).

OS Kaplan–Meier curves were generated for patient subgroups of sarcopenia based on the four above-described categorization methods: lb-SMI, cs-SMI, gt-SMI and bt-SMI low vs. high.

Chi-square and *p* values were obtained from log-rank test in the survival analysis and the most significant categorization method of SMI was selected to be assessed in a multivariate Cox-regression model for OS with known prognostic factors that included smoking habit, HPV status, ECOG PS and mGPS [21]. Calculations were carried out for 95% confidence intervals (CIs) for the estimated hazard ratios (HRs).

Differences in prevalence of sarcopenia between patient subgroups were assessed using classic chi-square test for categorical variables.

All analyses were performed using the R software v.4.0.3 and MedCalc software version 20.006. All tests were considered statistically significant for two-tail *p* values < 0.05.

## 3. Results

One-hundred-fifteen patients (median age 65 years, range 35 to 86 years, 24 females, 91 males) were included in the present analysis. Fifty-three patients (46%) had oropharyngeal cancer. And 72 (63%) patients received platinum-based RT while 43 (37%) received a Cisplatin-5FU-based first-line treatment with anti-EGFR or anti-PD-1 agent (36 and 7, respectively). Table 1 depicts the main patients’ characteristics. The OS endpoint was reached in 43 patients, and the median follow-up of surviving patients was 29.1 months (0.5 to 182 months). The median OS (mOS) of the entire cohort was 82.83 months (95% CI 35.75 to 106.58).

The median SMI in the cohort was 36.15 cm^2^/m^2^, ranging from 18.99 cm^2^/m^2^ to 53.99 cm^2^/m^2^.

**Figure 2:**
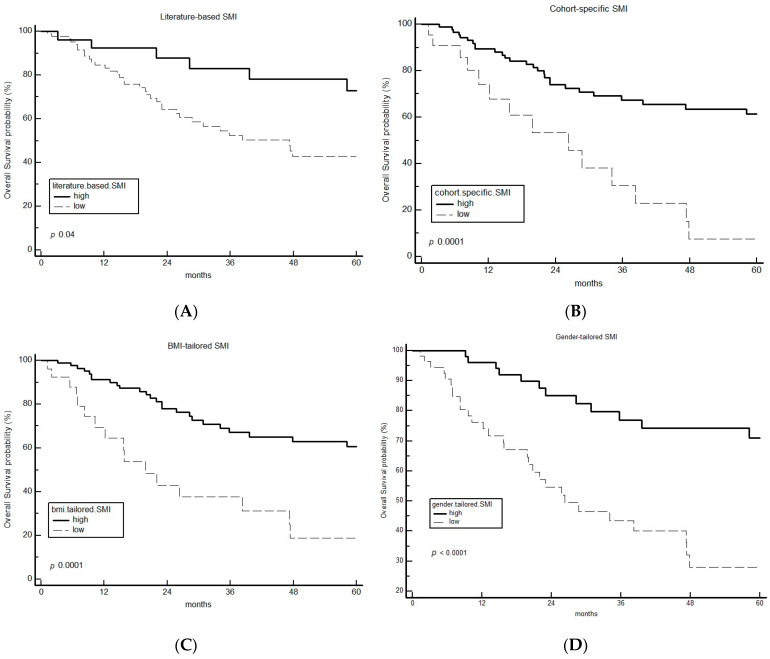
Kaplan–Meier curve of OS for the entire population selected for sarcopenia by using literature [14] (**A**), cohort-specific (obtained through maximally selected rank statistic) (**B**) and BMI- (**C**) and gender- (**D**) tailored SMI cut-offs. BMI: body mass index; OS: overall survival; SMI: sarcopenic mass index.

Taking into consideration the lb-SMI cutoff (43.2 cm^2^/m^2^) [16], 87 out of 115 patients (75.7%) were considered sarcopenic. Sarcopenic lb-SMI was associated with impaired survival (24-month OS rate 64% vs. 88% for lb-SMI low vs. high, respectively; hazard ratio (HR) 1.96; 95% confidence interval (CI) 1.03 to 3.74; *p* 0.04) (Figure 2A).

According to the maximally selected rank statistics for OS, the SMI cut-off in our cohort (cs-SMI cutoff) was 31.50 cm^2^/m^2^. A total of 22 out of 115 (19%) patients were sarcopenic according to the cs-SMI. The 24-month OS rate for cs-SMI low versus high was 53% vs. 74%, respectively (HR 6.61, 95%CI 2.65 to 16.50, *p* 0.0001) (Figure 2B).

The bt-SMI and gt-SMI cut-offs were obtained using a stratified maximally selected rank statistics and were 34.73 cm^2^/m^2^ for non-overweight (BMI < 25) patients, 27.32 cm^2^/m^2^ for overweight (BMI > 25) patients, 34.37 cm^2^/m^2^ for men and 46.02 cm^2^/m^2^ for women. Patients with SMI below the reference cut-off value according to the relevant category were classified as having sarcopenia.

According to bt-SMI and gt-SMI, 26 (23%) and 55 (48%) patients were found to be sarcopenic, respectively.

Of note, sarcopenia was more prevalent in females (20 out of 24 (83%)) than in males (35 out of 91 (39%)) according to the gt-SMI (chi-square *p*-value, 0.0001).

Sarcopenia according to gt-SMI was demonstrated to have a remarkably powerful prognostic effect (24-month OS rate 55% vs. 85% for gt-SMI low vs. high, respectively; HR 3.96 (95% CI 2.12 to 7.38) *p* < 0.0001 (Figure 2D)). Bt-SMI had a prognostic impact similar to that of cs-SMI (Figure 2C).

Twenty-four-month OS rate, univariate HR with 95% CI and *p* values, and chi-square statistics for the four SMI categorization methods are summarized in Table 2.

**Figure 3:**
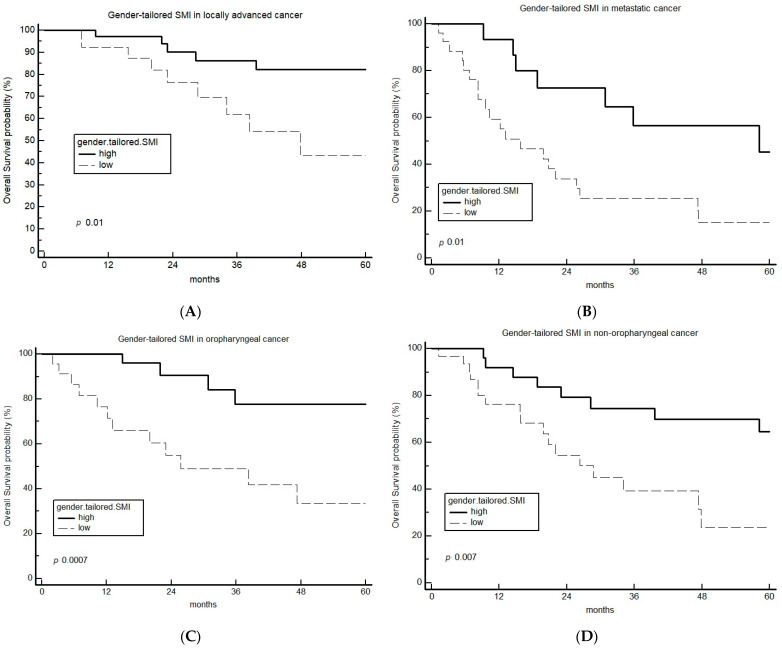
Kaplan–Meier curves of OS according to gender-tailored SMI sarcopenic assessment in locally advanced CRT-treated (**A**) and metastatic 1st-line-treated (**B**) patients and in oropharyngeal (**C**) and non-oropharyngeal (**D**) primary site. CRT: chemoradiotherapy; OS: overall survival; SMI: skeletal muscle index.

The prognostic value of the gt-SMI was confirmed both in patients with locally advanced disease treated with definitive chemoradiotherapy [HR 4.18, 95%CI 1.36 to 12.85, *p* 0.01] (Figure 3A) and in metastatic patients treated with CDDP-5FU-based first-line treatment [HR 2.63, 95%CI 1.26 to 5.49, *p* 0.01] (Figure 3B) and in both oropharyngeal and non-oropharyngeal cancer patients (HR 5.56 (95%CI 2.07 to 14.96), *p* 0.0007; and HR 3.04 (95%CI 1.36 to 6.81), *p* 0.007, respectively) (Figure 3C and Figure 3D, respectively).

To investigate whether gt-SMI remained an independent prognostic factor for OS when other HNSCC-specific prognostic factors (KPS, smoking habit, mGPS, HPV status and treatment setting) were considered, we fitted a multivariate Cox regression model (Table 3). When all the variables were entered into the model, gt-SMI sarcopenia was demonstrated to be an independent prognostic factor together with HPV positivity and treatment setting (HR for gt-SMI low vs. high 3.62 (95%CI 1.78 to 7.37), *p* 0.0004 (Table 3)).

## 4. Discussion

Cancer cachexia, or cancer sarcopenia, is a syndrome that comprises loss of both body weight and muscle mass. In HNSCC patients, it might accompany the disease course for a long time and emerge as a true chronic illness. Moreover, it is often linked to poor prognosis and worse treatment tolerability. The prognostic role of sarcopenia in HNSCC patient candidates for local treatment has been highlighted by two recent meta-analyses demonstrating that sarcopenia represents a poor prognostic factor in patients with localized cancer treated with surgery and/or radiotherapy [25,26].

Sarcopenia assessment is important, especially when cachexia is subclinical [27], and in certain settings, it has a stronger prognostic effect than the primary tumor site and patient performance status [28]. Furthermore, it can be present independently of the basal BMI [28].

CT scan is a feasible alternative to study and measure muscle mass. Although the L3-CT scan slice is the most commonly applied diagnostics for this purpose [29], it is not always available in HNSCC patients, since most scans only report on the cervicofacial area, without considering a whole-body CT scan [30]. In our analysis, C3-slice was used to calculate SMI in accordance with previous research, reporting a strong correlation between the measurement of sarcopenia at the cervical and the lumbar level [23].

In previous works [31,32,33,34,35], SMI threshold values obtained from non-HNSCC patients [18] were applied in HNSCC patients. However, due to the lack of consensus on the use of cut-off values of sarcopenia and the unique characteristics of each patient group, we are in favor of using a disease-specific one.

Wendrich et al. for the first time identified an HNSCC-specific cut-off of sarcopenia by analyzing a cohort of 112 locally advanced HNSCC [16]. Specifically, they demonstrated that SMI > 43.2 cm^2^/m^2^ could predict the dose-limiting toxicity of standard chemotherapy [16]. In our work, we focused on the potential association between sarcopenia as measured using SMI and survival outcome. We observed that 31.50 cm^2^/m^2^ was the SMI cut-off value with the greatest discrimination power in terms of OS in our cohort (cs-SMI). Based on our threshold value of sarcopenia, only about one quarter of patients who would have been defined as sarcopenic according to the ‘toxicity’ SMI cut-off by Wendrich et al. [16] were actually found sarcopenic according to the cs-SMI. The lower cutoff for survival deterioration simply indicates that the muscle mass required to be lost before having a significant impact on overall survival is higher than that posing the risk for toxicity.

Considering that skeletal muscle mass can differ in specific patient subgroups, we looked at differences in SMI cut-offs based on BMI (< vs. ≥25) and gender (male vs. female).

BMI is a simple tool, representative of adipose tissue, obtainable at baseline visit. Sarcopenia has been demonstrated to have a negative impact on prognosis both in severely cachectic underweight patients with a contemporary loss of fat and lean tissue [31,34,36] and in sarcopenic obese patients [18,37,38]. Lean muscle tissue can vary according to the BMI [31]. Myo-steatosis, defined as the lipid content of skeletal muscle, is increased in obesity, and this might influence the CT-scan based measurement of the lean muscle tissue [39]. Sarcopenic assessment by using SMI cut-offs adjusted for BMI could therefore partly overcome this risk. Indeed, we found that overweight patients may tolerate a higher muscle loss before survival deterioration as compared to non-overweight patients (SMI cut-off of 27.32 and 34.73, respectively), possibly because of a higher energy reserve (so-called obesity paradox) [18].

Gender might be implicated in sarcopenia assessment because of the difference in proportion of specific body components per body surface area between males and females [17]. In an era of personalized medicine, physiological differences between men and women, as well as the potential role played by sex-hormones in tumor biology, should be taken into account [40]. Importantly, a mounting body of evidence is supporting our hypothesis in terms of outcome, treatment benefit and adverse events between men and women in non-sex-related cancers [41]. Discrepancies in gene expression in skeletal muscle tissue as well as in thyroid hormone, estrogen and testosterone levels might result in a different fiber-type composition and contractility between men and women [42]. Moreover, Park et al., by analyzing the intestinal microbiota using 16S rRNA gene sequencing in more than 1000 Korean patients, showed that sex-specific gut microbiome diversity might be associated with different skeletal mass tissue amounts [43]. Finally, recent reports conducted on community-dwelling adults have evidenced that different risk factors might lead to sarcopenia in men and women, suggesting that gender-tailored preventing activities (particularly resistance training versus nutritional support) should be designed [44]. These data suggest that gender should be considered when muscle tissue measurement is performed.

Only few studies have previously taken into account gender to define sarcopenia in HNSCC patients [45,46,47,48,49]. However, all of them focused on treatment toxicity and did not evaluate survival endpoints. Moreover, threshold values were identified using quintiles and not cut-off optimization methods [45,46,47]. We focused on identifying specific cutoffs for patients with HNSCC undergoing definitive chemo-radiotherapy or first-line bio-chemotherapy by using distinct thresholds for males and females, since these HNSCC populations might have different risk factors and cancer biology.

Also, Ye et al. [50] recently developed an image-based deep learning platform for SMI calculation to evaluate associations with survival and treatment toxicity outcomes and used separate thresholds for males and females. However, they used literature-based cutoffs [19] established on a mixed population of 250 patients with different types of cancer (mostly colon, rectum and other gastrointestinal or respiratory tract sites,). We consider this a clear limitation given the heterogeneity between different cancer types. The novelty of our study relies on the improved prognostication obtained by tuning the SMI cutoff based on gender and a specific cancer setting.

The high prognostic effect of gt-SMI found in our study (*p* value < 0.0001) provides support for the interaction between body composition, sex and lean tissue. Interestingly, the prevalence of sarcopenia assessed by gt-SMI cut-off is consistent with that reported by Surov et al. in a recent meta-analysis including 7704 HNSCC patients [7]. We found a superior SMI threshold to define survival-associated sarcopenia in women. No clear explanation could be identified for this finding. A higher sensitivity to muscle mass reduction impacting on survival can be hypothesized in women, but further investigation in this respect is highly warranted.

The prognostic role of sarcopenia defined by gt-SMI was observed regardless of the treatment setting and the primary tumor site (Figure 3). Moreover, gt-SMI retained its independent effect on survival when included in a multivariable model with other well-known HNSCC-specific prognostic factors, including inflammatory biomarkers such as mGPS. Further analyses are needed to confirm that sarcopenia has a prognostic role independently of systemic inflammation, although the strong link between these two conditions has been repeatedly reported in cancer and other chronic diseases [21].

Early identification of sarcopenia and the application of effective rehabilitation programs would be of great value for HNSCC patients. Previous works have shown that physical exercise focused on resistance activity to increase strength and muscle mass, and nutritional interventions such as the increase in protein intake up to 2 g a day and the supplementation of essential amino acids and ß-hydroxy ß-methylbutyric acid are able to improve the sarcopenic status [4,48,49].

Chemotherapy-dose-limiting toxicities reported in sarcopenic patients [16] also deserve special attention with a need to quickly recognize this status to eventually tailor drug dosing [51]. For example, cisplatin, a key chemotherapeutic drug used in HNSCC, is partly absorbed in the muscle tissue where it is metabolized with mild toxic effects [52]. Chargi et al., by analyzing cisplatin pharmacokinetics in 45 HNSCC patients, confirmed that patients with lower muscle mass were more prone to cisplatin-related toxicity because of the higher drug concentration in non-muscle tissues, where it exerted toxic effects [53].

The retrospective nature of our work is a major limitation, but the results should generate a hypothesis in prospectively evaluating our findings in a more defined patient population. Moreover, we included a mixed population of locally advanced and metastatic HNSCC patients and a distinct analysis with adequate sample size of these two subgroups of patients would be highly desirable. A further drawback is that the cross-sectional area (CSA) to measure SMI was manually derived by reviewing available CT scans. An automated measurement of CSA would be preferable to minimize operator-dependent errors in the analytic procedure. Finally, external validation of the identified SMI cut-offs in larger cohorts is highly recommended.

Sarcopenia has been demonstrated to be strongly related to the immune system [48,54]. Several studies have confirmed a negative prognostic role of sarcopenia in cancer patients treated with immune-checkpoint inhibitors, probably due to increased release of proinflammatory cytokines dysregulating T-cell activity [55]. Further studies are needed to evaluate if sarcopenia could directly affect PD-L1 expression in HNSCC patients, considering the potential therapeutic implications.

## 5. Conclusions

Our study confirmed the negative correlation between sarcopenia, derived from CT-scans of the head and neck region, and survival in an HNSCC patient cohort, treated with standard chemotherapy +/− RT. Gender should be taken into account in the assessment of sarcopenia, to allow adequate preventive programs as well as to evaluate personalized treatment plans.

## Figures and Tables

**Table 1:** Baseline patients’ characteristics.

Variable *	Locally Advanced Disease	Metastatic Disease
	No. of patients [%]	No. of patients [%]
Gender		
male	59 [81.94]	32 [74.41]
females	13 [18.06]	11 [25.59]
Median age (range) in years	66 (40–86)	64 (35–83)
Body Mass Index		
<25 Kg/m^2^	43 [59.72]	24 [55.81]
≥25 Kg/m^2^	29 [40.28]	19 [44.19]
Tumor stage		
III	72 [100]	-
IV	-	43 [100]
Tumor site		
Oropharyngeal HPV-positive	18 [25.00]	9 [20.93]
Oropharyngeal HPV-negative	16 [22.22]	10 [23.25]
Non-oropharyngeal	38 [52.78]	24 [55.82]
Smoking habits		
former/current	63 [87.50]	33 [76.75]
never	9 [12.50]	10 [23.25]
Alcohol consumption		
yes	53 [73.61]	14 [32.56]
no	19 [26.39]	29 [67.44]
Charlson Comorbidity Score		
<9	57 [79.17]	30 [69.77]
≥9	15 [20.83]	13 [30.23]
Treatment		36 [83.75]
Cisplatin-5FU-Cetuximab first-line	-	7 [16.28]
Cisplatin-5FU-Pembrolizumab first-line	-	-
Cisplatin-based CRT	72 [100]	
mGPS		
0	19 [26.39]	12 [27.91]
1–2	53 [73.61]	31 [72.09]
KPS		
100–90	53 [73.61]	26 [60.46]
≤80	19 [26.39]	17 [39.54]

5FU: 5-Fluoruracil; CDDP: cisplatin; CRT: chemoradiotherapy; HPV: Human Papilloma Virus; KPS: Karnofsky Performance Status; mGPS: modified Glasgow Prognostic Score, which is based on the number of the following adverse factors that are present: C-reactive protein > 10 mg/L, albumin < 3.5 g/dL. * values refer to n. of patients [percentage], unless otherwise specified (e.g., median age).

**Table 2:** Univariate Cox-regression analysis for overall survival, chi-square and *p*-values from Log Rank test for comparison between survival curves obtained by using literature, cohort-specific, and BMI- and gender-tailored SMI cut-offs in HNSCC patients.

SMI	*p* Value	24-Month-OS (%)	Univariate HR (95%CI)
Literature-based-SMI low vs. high	0.04	64 vs. 88	1.96 (1.03 to 3.74)
Cohort-specific-SMI low vs. high	0.0001	53 vs. 74	6.61 (2.65 to 16.50)
BMI-tailored SMI low vs. high	0.0001	43 vs. 78	5.52 (2.40 to 12.73)
Gender-tailored SMI low vs. high	<0.0001	55 vs. 85	3.96 (2.12 to7.38)

BMI: body mass index; HNSCC: head and neck squamous cell carcinoma; SMI: sarcopenic mass index.

**Table 3:** Multivariate Cox-regression analysis of several sarcopenic-related prognostic factors for overall survival of HNSCC patients.

Prognostic Factor	HR	95% CI	*p* Value
Gender-tailored SMI (low vs. high)	3.62	1.78 to 7.37	0.0004
HPV status (positive vs. negative)	0.22	0.09 to 0.52	0.0006
KPS (≤80 vs. 100–90)	0.87	0.46 to 1.67	0.68
mGPS (0 vs. 1–2)	0.54	0.25 to 1.17	0.12
smoking habit (current or former vs. never)	1.30	0.59 to 2.83	0.51
Line of treatment (CRT vs. first-line treatment)	4.79	2.47 to 9.29	<0.0001

BMI: body mass index; CI: confidence interval; CRT: chemoradiotherapy; HNSCC: head and neck squamous cell carcinoma; HR: Hazard ratio; KPS: Karnofsky Performance Status; mGPS: modified Glasgow Prognostic Score; SMI: Sarcopenic Mass Index.

## Data Availability

The data presented in this study are available on request from the corresponding author. The data are not publicly available due to privacy regulations in force at our hospital.

## References

[1] Sung H., Ferlay J., Siegel R.L., Laversanne M., Soerjomataram I., Jemal A., Bray F. (2021). Global Cancer Statistics 2020: GLOBOCAN Estimates of Incidence and Mortality Worldwide for 36 Cancers in 185 Countries. CA Cancer J. Clin..

[2] Mody M.D., Rocco J.W., Yom S.S., Haddad R.I., Saba N.F. (2021). Head and neck cancer. Lancet.

[3] Chow L.Q.M. (2020). Head and Neck Cancer. N. Engl. J. Med..

[4] Bauer J., Morley J.E., Schols A.M.W.J., Ferrucci L., Cruz-Jentoft A.J., Dent E., Baracos V.E., Crawford J.A., Doehner W., Heymsfield S.B. (2019). Sarcopenia: A Time for Action. An SCWD Position Paper. J. Cachexia Sarcopenia Muscle.

[5] Nipp R.D., Fuchs G., El-Jawahri A., Mario J., Troschel F.M., Greer J.A., Gallagher E.R., Jackson V.A., Kambadakone A., Hong T.S. (2018). Sarcopenia Is Associated with Quality of Life and Depression in Patients with Advanced Cancer. Oncologist.

[6] Angulo J., El Assar M., Rodríguez-Mañas L. (2016). Frailty and sarcopenia as the basis for the phenotypic manifestation of chronic diseases in older adults. Mol. Asp. Med..

[7] Surov A., Wienke A. (2022). Prevalence of sarcopenia in patients with solid tumors: A meta-analysis based on 81,814 patients. JPEN J. Parenter. Enteral Nutr..

[8] Pamoukdjian F., Bouillet T., Lévy V., Soussan M., Zelek L., Paillaud E. (2018). Prevalence and predictive value of pre-therapeutic sarcopenia in cancer patients: A systematic review. Clin. Nutr..

[9] Morelli C., Formica V., Patrikidou A., Rofei M., Shiu K.K., Riondino S., Argirò R., Floris R., Ferlosio A., Orlandi A. (2022). Nutritional index for immune-checkpoint inhibitor in patients with metastatic gastro-esophageal junction/gastric cancer. J. Gastrointest. Oncol..

[10] Gouez M., Delrieu L., Bouleuc C., Girard N., Raynard B., Marchal T. (2022). Association between Nutritional Status and Treatment Response and Survival in Patients Treated with Immunotherapy for Lung Cancer: A Retrospective French Study. Cancers.

[11] Li S., Wang T., Tong G., Li X., You D., Cong M. (2021). Prognostic Impact of Sarcopenia on Clinical Outcomes in Malignancies Treated With Immune Checkpoint Inhibitors: A Systematic Review and Meta-Analysis. Front. Oncol..

[12] Baumgartner R.N., Koehler K.M., Gallagher D., Romero L., Heymsfield S.B., Ross R.R., Garry P.J., Lindeman R.D. (1998). Epidemiology of sarcopenia among the elderly in New Mexico. Am. J. Epidemiol..

[13] Shen W., Punyanitya M., Wang Z., Gallagher D., St-Onge M.P., Albu J., Heymsfield S.B., Heshka S. (2004). Total body skeletal muscle and adipose tissue volumes: Estimation from a single abdominal cross-sectional image. J. Appl. Physiol. (1985).

[14] Mourtzakis M., Prado C.M., Lieffers J.R., Reiman T., McCargar L.J., Baracos V.E. (2008). A practical and precise approach to quantification of body composition in cancer patients using computed tomography images acquired during routine care. Appl. Physiol. Nutr. Metab..

[15] Wong A., Zhu D., Kraus D., Tham T. (2021). Radiologically Defined Sarcopenia Affects Survival in Head and Neck Cancer: A Meta-Analysis. Laryngoscope.

[16] Wendrich A.W., Swartz J.E., Bril S.I., Wegner I., de Graeff A., Smid E.J., de Bree R., Pothen A.J. (2017). Low skeletal muscle mass is a predictive factor for chemotherapy dose-limiting toxicity in patients with locally advanced head and neck cancer. Oral Oncol..

[17] Yoon J.K., Jang J.Y., An Y.S., Lee S.J. (2021). Skeletal muscle mass at C3 may not be a strong predictor for skeletal muscle mass at L3 in sarcopenic patients with head and neck cancer. PLoS ONE.

[18] Prado C.M., Baracos V.E., McCargar L.J., Mourtzakis M., Mulder K.E., Reiman T., Butts C.A., Scarfe A.G., Sawyer M.B. (2007). Body composition as an independent determinant of 5-fluorouracil-based chemotherapy toxicity. Clin. Cancer Res..

[19] Prado C.M., Lieffers J.R., McCargar L.J., Reiman T., Sawyer M.B., Martin L., Baracos V.E. (2008). Prevalence and clinical implications of sarcopenic obesity in patients with solid tumours of the respiratory and gastrointestinal tracts: A population-based study. Lancet Oncol..

[20] Charlson M.E., Pompei P., Ales K.L., MacKenzie C.R. (1987). A new method of classifying prognostic comorbidity in longitudinal studies: Development and validation. J. Chronic Dis..

[21] Hacker U.T., Hasenclever D., Baber R., Linder N., Busse H., Obermannova R., Zdrazilova-Dubska L., Valik D., Lordick F. (2022). Modified Glasgow prognostic score (mGPS) is correlated with sarcopenia and dominates the prognostic role of baseline body composition parameters in advanced gastric and esophagogastric junction cancer patients undergoing first-line treatment from the phase III EXPAND trial. Ann. Oncol..

[22] (2000). Obesity: Preventing and managing the global epidemic. Report of a WHO consultation. World Health Organ. Tech. Rep. Ser..

[23] Swartz J.E., Pothen A.J., Wegner I., Smid E.J., Swart K.M., de Bree R., Leenen L.P., Grolman W. (2016). Feasibility of using head and neck CT imaging to assess skeletal muscle mass in head and neck cancer patients. Oral Oncol..

[24] Schulgen G., Lausen B., Olsen J.H., Schumacher M. (1995). Outcome-oriented cutpoints in analysis of quantitative exposures. Am. J. Epidemiol..

[25] Takenaka Y., Takemoto N., Oya R., Inohara H. (2021). Prognostic impact of sarcopenia in patients with head and neck cancer treated with surgery or radiation: A meta-analysis. PLoS ONE.

[26] Findlay M., White K., Stapleton N., Bauer J. (2021). Is sarcopenia a predictor of prognosis for patients undergoing radiotherapy for head and neck cancer? A meta-analysis. Clin. Nutr..

[27] Bozzetti F. (2017). Forcing the vicious circle: Sarcopenia increases toxicity, decreases response to chemotherapy and worsens with chemotherapy. Ann. Oncol..

[28] Martin L., Birdsell L., Macdonald N., Reiman T., Clandinin M.T., McCargar L.J., Murphy R., Ghosh S., Sawyer M.B., Baracos V.E. (2013). Cancer cachexia in the age of obesity: Skeletal muscle depletion is a powerful prognostic factor, independent of body mass index. J. Clin. Oncol..

[29] Kazemi-Bajestani S.M., Mazurak V.C., Baracos V. (2016). Computed tomography-defined muscle and fat wasting are associated with cancer clinical outcomes. Semin. Cell Dev. Biol..

[30] Machiels J.P., René Leemans C., Golusinski W., Grau C., Licitra L., Gregoire V., EHNS Executive Board (2020). Squamous cell carcinoma of the oral cavity, larynx, oropharynx and hypopharynx: EHNS-ESMO-ESTRO Clinical Practice Guidelines for diagnosis, treatment and follow-up. Ann. Oncol..

[31] Grossberg A.J., Chamchod S., Fuller C.D., Mohamed A.S., Heukelom J., Eichelberger H., Kantor M.E., Hutcheson K.A., Gunn G.B., Garden A.S. (2016). Association of Body Composition With Survival and Locoregional Control of Radiotherapy-Treated Head and Neck Squamous Cell Carcinoma. JAMA Oncol..

[32] Zwart A.T., van der Hoorn A., van Ooijen P.M.A., Steenbakkers R.J.H.M., de Bock G.H., Halmos G.B. (2019). CT-measured skeletal muscle mass used to assess frailty in patients with head and neck cancer. J. Cachexia Sarcopenia Muscle.

[33] Jung A.R., Roh J.L., Kim J.S., Kim S.B., Choi S.H., Nam S.Y., Kim S.Y. (2019). Prognostic value of body composition on recurrence and survival of advanced-stage head and neck cancer. Eur. J. Cancer.

[34] Willemsen A.C.H., Hoeben A., Lalisang R.I., Van Helvoort A., Wesseling F.W.R., Hoebers F., Baijens L.W.J., Schols A.M.W.J. (2020). Disease-induced and treatment-induced alterations in body composition in locally advanced head and neck squamous cell carcinoma. J. Cachexia Sarcopenia Muscle.

[35] Thureau S., Lebret L., Lequesne J., Cabourg M., Dandoy S., Gouley C., Lefebvre L., Mallet R., Mihailescu S.D., Moldovan C. (2021). Prospective Evaluation of Sarcopenia in Head and Neck Cancer Patients Treated with Radiotherapy or Radiochemotherapy. Cancers.

[36] Richey L.M., George J.R., Couch M.E., Kanapkey B.K., Yin X., Cannon T., Stewart P.W., Weissler M.C., Shores C.G. (2007). Defining cancer cachexia in head and neck squamous cell carcinoma. Clin. Cancer Res..

[37] Caan B.J., Cespedes Feliciano E.M., Prado C.M., Alexeeff S., Kroenke C.H., Bradshaw P., Quesenberry C.P., Weltzien E.K., Castillo A.L., Olobatuyi T.A. (2018). Association of Muscle and Adiposity Measured by Computed Tomography With Survival in Patients With Nonmetastatic Breast Cancer. JAMA Oncol..

[38] Wang K., Yu X.H., Tang Y.J., Tang Y.L., Liang X.H. (2019). Obesity: An emerging driver of head and neck cancer. Life Sci..

[39] Findlay M., White K., Brown C., Bauer J.D. (2021). Nutritional status and skeletal muscle status in patients with head and neck cancer: Impact on outcomes. J. Cachexia Sarcopenia Muscle.

[40] Costa A.R., Lança de Oliveira M., Cruz I., Gonçalves I., Cascalheira J.F., Santos C.R.A. (2020). The Sex Bias of Cancer. Trends Endocrinol. Metab..

[41] Haupt S., Caramia F., Klein S.L., Rubin J.B., Haupt Y. (2021). Sex disparities matter in cancer development and therapy. Nat. Rev. Cancer..

[42] Haizlip K.M., Harrison B.C., Leinwand L.A. (2015). Sex-based differences in skeletal muscle kinetics and fiber-type composition. Physiology.

[43] Park C.H., Lee E.J., Kim H.L., Lee Y.T., Yoon K.J., Kim H.N. (2022). Sex-specific associations between gut microbiota and skeletal muscle mass in a population-based study. J. Cachexia Sarcopenia Muscle.

[44] Choe H.J., Cho B.L., Park Y.S., Roh E., Kim H.J., Lee S.G., Kim B.J., Kim M., Won C.W., Park K.S. (2022). Gender differences in risk factors for the 2 year development of sarcopenia in community-dwelling older adults. J. Cachexia Sarcopenia Muscle.

[45] van Rijn-Dekker M.I., van den Bosch L., van den Hoek J.G.M., Bijl H.P., van Aken E.S.M., van der Hoorn A., Oosting S.F., Halmos G.B., Witjes M.J.H., van der Laan H.P. (2020). Impact of sarcopenia on survival and late toxicity in head and neck cancer patients treated with radiotherapy. Radiother. Oncol..

[46] Zwart A.T., Pörtzgen W., van Rijn-Dekker I., Sidorenkov G.A., Dierckx R.A.J.O., Steenbakkers R.J.H.M., Wegner I., van der Hoorn A., de Bock G.H., Halmos G.B. (2022). Sex-Specific Cut-Off Values for Low Skeletal Muscle Mass to Identify Patients at Risk for Treatment-Related Adverse Events in Head and Neck Cancer. J. Clin. Med..

[47] Karavolia E., van Rijn-Dekker M.I., Van den Bosch L., van den Hoek J.G.M., Oldehinkel E., Meijer T.W.H., Halmos G.B., Witjes M.J.H., Oosting S.F., van der Hoorn A. (2022). Impact of sarcopenia on acute radiation-induced toxicity in head and neck cancer patients. Radiother. Oncol..

[48] Nelke C., Dziewas R., Minnerup J., Meuth S.G., Ruck T. (2019). Skeletal muscle as potential central link between sarcopenia and immune senescence. EBioMedicine.

[49] Cruz-Jentoft A.J., Landi F., Schneider S.M., Zúñiga C., Arai H., Boirie Y., Chen L.K., Fielding R.A., Martin F.C., Michel J.P. (2014). Prevalence of and interventions for sarcopenia in ageing adults: A systematic review. Report of the International Sarcopenia Initiative (EWGSOP and IWGS). Age Ageing.

[50] Ye Z., Saraf A., Ravipati Y., Hoebers F., Catalano P.J., Zha Y., Zapaishchykova A., Likitlersuang J., Guthier C., Tishler R.B. (2023). Development and Validation of an Automated Image-Based Deep Learning Platform for Sarcopenia Assessment in Head and Neck Cancer. JAMA Netw. Open.

[51] Silvestris N., Argentiero A., Natalicchio A., D’Oronzo S., Beretta G.D., Acquati S., Adinolfi V., Di Bartolo P., Danesi R., Faggiano A. (2021). Antineoplastic dosing in overweight and obese cancer patients: An Associazione Italiana Oncologia Medica (AIOM)/Associazione Medici Diabetologi (AMD)/Società Italiana Endocrinologia (SIE)/Società Italiana Farmacologia (SIF) multidisciplinary consensus position paper. ESMO Open.

[52] Karasawa T., Steyger P.S. (2015). An integrated view of cisplatin-induced nephrotoxicity and ototoxicity. Toxicol. Lett..

[53] Chargi N., Molenaar-Kuijsten L., Huiskamp L.F.J., Devriese L.A., de Bree R., Huitema A.D.R. (2022). The association of cisplatin pharmacokinetics and skeletal muscle mass in patients with head and neck cancer: The prospective PLATISMA study. Eur. J. Cancer.

[54] Wilson D., Jackson T., Sapey E., Lord J.M. (2017). Frailty and sarcopenia: The potential role of an aged immune system. Ageing Res. Rev..

[55] Bilen M.A., Martini D.J., Liu Y., Shabto J.M., Brown J.T., Williams M., Khan A.I., Speak A., Lewis C., Collins H. (2020). Combined Effect of Sarcopenia and Systemic Inflammation on Survival in Patients with Advanced Stage Cancer Treated with Immunotherapy. Oncologist.

